# SeqAssist: a novel toolkit for preliminary analysis of next-generation sequencing data

**DOI:** 10.1186/1471-2105-15-S11-S10

**Published:** 2014-10-21

**Authors:** Yan Peng, Andrew S Maxwell, Natalie D Barker, Jennifer G Laird, Alan J Kennedy, Nan Wang, Chaoyang Zhang, Ping Gong

**Affiliations:** 1 School of Computing, University of Southern Mississippi, Hattiesburg, MS 39406, USA; 2 Badger Technical Services, LLC, San Antonio, TX 78216, USA; 3 Environmental Laboratory, U.S. Army Engineer Research and Development Center, Vicksburg, MS 39180, USA

**Keywords:** SeqAssist, next generation sequencing (NGS), sequencing data analysis, genome-wide coverage, breadth, depth, evenness, genome (re-)sequencing, RNA-Seq, FASTQ, BWA-MEM.

## Abstract

**Background:**

While next-generation sequencing (NGS) technologies are rapidly advancing, an area that lags behind is the development of efficient and user-friendly tools for preliminary analysis of massive NGS data. As an effort to fill this gap to keep up with the fast pace of technological advancement and to accelerate data-to-results turnaround, we developed a novel software package named SeqAssist ("Sequencing Assistant" or SA).

**Results:**

SeqAssist takes NGS-generated FASTQ files as the input, employs the BWA-MEM aligner for sequence alignment, and aims to provide a quick overview and basic statistics of NGS data. It consists of three separate workflows: (1) the SA_RunStats workflow generates basic statistics about an NGS dataset, including numbers of raw, cleaned, redundant and unique reads, redundancy rate, and a list of unique sequences with length and read count; (2) the SA_Run2Ref workflow estimates the breadth, depth and evenness of genome-wide coverage of the NGS dataset at a nucleotide resolution; and (3) the SA_Run2Run workflow compares two NGS datasets to determine the redundancy (overlapping rate) between the two NGS runs. Statistics produced by SeqAssist or derived from SeqAssist output files are designed to inform the user: whether, what percentage, how many times and how evenly a genomic locus (i.e., gene, scaffold, chromosome or genome) is covered by sequencing reads, how redundant the sequencing reads are in a single run or between two runs. These statistics can guide the user in evaluating the quality of a DNA library prepared for RNA-Seq or genome (re-)sequencing and in deciding the number of sequencing runs required for the library. We have tested SeqAssist using a synthetic dataset and demonstrated its main features using multiple NGS datasets generated from genome re-sequencing experiments.

**Conclusions:**

SeqAssist is a useful and informative tool that can serve as a valuable "assistant" to a broad range of investigators who conduct genome re-sequencing, RNA-Seq, or *de novo *genome sequencing and assembly experiments.

## Background

High throughput next-generation sequencing (NGS) technologies are capable of generating massive amounts of data in the form of paired-end or single-end reads with either fixed or variable lengths. The size of data files is often in the magnitude of mega- or giga-bytes (up to 1000 giga base pairs or Gb in a single sequencing run) and is likely to increase further in the years to come. While sequencing costs have dropped precipitously and sequencing speed and efficiency have risen exponentially, development of computational tools for preliminary analysis of these gigantic datasets have lagged behind data generation. Hence, there is an increasing demand for efficient and user-friendly programs for preliminary sequencing data analysis.

Currently, there are four commercially predominant NGS platforms, including Illumina/Solexa, Roche/454, ABI/SOLiD and ABI/Ion Torrent [1,2]. These massively parallel DNA sequencing technologies have been applied to transcriptome sequencing (RNA-Seq), *de novo *genome sequencing, and genome re-sequencing. RNA-Seq is a widely used approach to transcriptomic profiling [3,4]. Two representative efforts in *de novo *genome sequencing are the Genome 10K project to obtain whole genome sequences for 10,000 vertebrate species [5-7] and the 5K Insect Genome Initiative (i5K) to sequence the genomes of 5,000 arthropod species [8,9]. Genome re-sequencing is an experimental procedure that involves sequencing individual organisms whose genome is already known [10]. As a new genomics approach, genome re-sequencing has been applied to a wide range of fundamental and applied biological research including genetics, evolution, biomedicine, human diseases and environmental health, with good examples being the 1000 Genomes Project [11] and the Cancer Genomes project [12].

Prior to in-depth analysis of NGS deep sequencing data, e.g., differential gene expression and alternative splicing analysis for RNA-Seq studies, structural variants identification for genome re-sequencing studies, and genome assembly for *de novo *genome sequencing studies, investigators are often concerned about the following issues: (1) basic statistics of a sequencing run such as total numbers of raw, cleaned, and unique reads as well as the degree of reads redundancy; (2) sequencing library quality, i.e., does the library truly represent the genome of the re-sequenced organism, and (3) the number of sequencing runs required, i.e., how many runs are necessary to get a full representation of the sequencing library or to suffice a *de novo *genome assembly. Although many tools such as Partek Genomics Suite, CLC Genomic Workbench, or noncommercial platforms like Galaxy and GenePattern are currently available and capable of indirectly addressing these issues, one would have to possess some basic bioinformatics training and script writing skills in order to manipulate and turn the generated results into useful and straightforward information that can be easily understood by an experimentalists. Motivated by filling this gap, the limitations of existing tools, and also driven by the demand for accelerating data-to-results turnaround, we have developed a novel toolkit named SeqAssist ("Sequencing Assistant", acronym: SA). SeqAssist specifically addresses the aforementioned three issues and provides investigators who conduct RNA-Seq, *de novo *genome sequencing or genome re-sequencing experiments with a quick overview and preliminary analysis of their NGS data.

## Implementation

**Figure 1:**
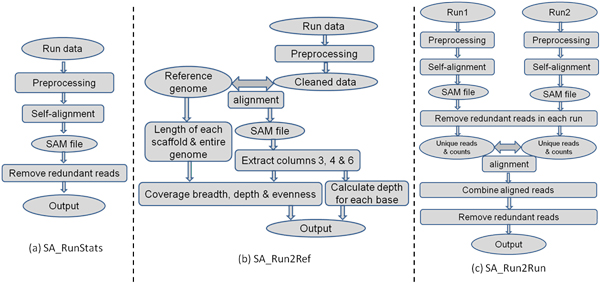
**SeqAssist (SA) workflows: (a) SA_RunStats, (b) SA_Run2Ref, and (c) SA_Run2Run**. The output of each workflow is described in details in the **Implementation **section.

SeqAssist was programmed using Perl with an additional Java-enabled Graphic User Interface to enhance efficiency and user-friendliness. It currently consists of three separate workflows: SA_RunStats, SA_Run2Ref and SA_Run2Run. SA_RunStats generates the basic statistics such as total number of raw and cleaned reads, length and copy number of unique sequences, and reads redundancy in a single sequencing run or a pooled dataset of several runs (see Figure 1a). The input of this workflow is a FASTQ-formatted sequencing data file. The data file is preprocessed to trim off adaptors and low quality read ends with a default cutoff of base-calling quality score (Q) at 20, followed by removal of N-containing reads. Then, the cleaned reads are aligned against each other using BWA-MEM (acronym for Burrows-Wheeler Aligner-Maximal Exact Match), one of the three Burrows-Wheeler Transform-based algorithms in the BWA software package [13,14]. BWA-MEM is a robust, fast and accurate aligner that supports paired-end reads, performs chimeric alignment, and tolerates sequencing errors (http://arxiv.org/abs/1303.3997v2). Based on the alignment information in the BWA-MEM-generated SAM (acronym for Sequence Alignment/Map format) file [15], the number of unique reads is counted and both identical and inclusive (i.e., redundant) reads are removed. Two reads are considered identical if they match 100% with each other and they are of equal length, while inclusive reads are defined as the sub-sequences of a longer read and only the longest read is kept as the unique read. The redundancy rate is calculated as the percentage of redundant reads in the total number of unique cleaned reads [see *Eq*. (1) for formula]. The output of this workflow includes the total numbers of raw, cleaned, redundant and unique reads, and the redundancy rate. Also included in the output is a tab-delimited plain text file that lists all unique sequences along with their length and read count (copy number). This file can be used to further infer gene expression levels if the run data is produced for an RNA-Seq experiment.

(1) $$\text{Redundancy rate}\left(\text{\%}\right)=\frac{\text{number of redundant reads}}{\text{total number of unique cleaned reads}}\times 100\text{\%}$$

SA_Run2Ref analyzes the breadth, depth and evenness of genome-wide coverage of an individual or pooled sequencing dataset at a nucleotide resolution. Coverage breadth is defined as the percentage of a reference sequence (i.e., gene, scaffold/chromosome, or entire genome) that is covered by sequencing reads [*Eq*. (2)]; coverage depth is defined as the average times a reference sequence is covered [*Eq*. (3)]; and coverage evenness is defined as the coefficient of variance of scaffold coverage breadth [*Eq*. (4)]. Therefore, outputs from SA_Run2Ref can inform what genomic loci are covered and how a genomic locus (gene), scaffold or the entire genome is covered. In the SA_Run2Ref workflow (Figure 1b), cleaned reads are aligned against the reference genome sequence, generating a SAM file. Information stored in columns 3, 4 and 6 for each alignment in the SAM file represents mandatory fields RNAME (reference sequence name), POS (1-based leftmost mapping position), and CIGAR (CIGAR string), respectively [15]. This information is extracted along with the length of each scaffold of reference genome to compute scaffold coverage breadth and depth and genome coverage evenness. These statistics are provided in the output files. The output also includes a plain-text file that records the coverage depth of each individual base on the entire genome. This file can be used as an input for genome browser tools to visualize coverage depth of any genomic regions. In case that the user conducts an RNA-Seq experiment and provides gene model sequences (instead of scaffold or chromosome sequences) as the input, the workflow will calculate coverage breadth and depth for each gene model. This information can be readily transformed into gene expression measurements.

(2) $$\text{Coverage breadth}\left(\text{\%}\right)=\frac{\text{number of reference bases mapped by sequencing reads}}{\text{length of the reference sequence in bases}}\times 100\text{\%}$$

(3) $$\text{Coverage depth}=\frac{\text{total number of bases mapped to the reference}}{\text{length of the reference sequence in bases}}$$

(4) $$\text{Coverage evenness}=\frac{\text{standard deviation of scaffold coverage breadth}}{\text{average scaffold coverage breadth}}$$

SA_Run2Run compares two separate sequencing datasets generated for the same or different DNA libraries, computes the basic statistics for each individual dataset, and estimates the redundancy rate between the two datasets. SA_Run2Run informs the user of the redundancy level both within each individual run and between two sequencing runs. Like the SA_RunStats workflow (Figure 1a), each input run dataset in the SA_Run2Run workflow (Figure 1c) independently goes through the same preprocessing, self-alignment and removing redundant reads steps to generate two new datasets containing unique cleaned sequences. Then, the two new datasets are aligned against each other using BWA-MEM, generating a new SAM file. After combining all aligned reads, reads common to both datasets (i.e., overlapping reads) are identified, the counts of redundant reads (identical or inclusive) are calculated for both overlapping and non-overlapping reads. The output statistics from SA_Run2Run include total numbers of raw reads, cleaned reads, and unique reads (after removing identical reads and inclusive reads), and numbers of total and unique overlapping reads. The redundancy rates within each dataset and between the two datasets can be further derived from these statistics. Similar to the SA_RunStats output, a list of unique sequences along with their length and count number is provided for each run. However, different from the SA_RunStats output, the list generated by SA_Run2Run is broken into two files, one containing overlapping reads and the other non-overlapping reads. To compare two paired-end sequencing runs, one has to run this workflow twice: Run1_R1.fastq vs. Run2_R1.fastq and Run1_R2.fastq vs. Run2_R2.fastq. The SA_Run2Run workflow intends to guide the user in deciding whether to perform more runs on a sequencing library by looking at the percentage of reads in a new run covered by the reads in a previous run or the pooled reads of multiple previous runs.

## Results and discussion

To test all SeqAssist workflows, a synthetic dataset was generated by (1) clipping 10 distinct fragments with a length of 150 bp at different loci of the *Escherichia coli *str. K-12 substr. MG1655l genome (NCBI Reference Sequence Accession No. NC_000913.3, available at http://www.ncbi.nlm.nih.gov/nuccore/556503834?report=fasta) to construct 10 artificial chromosomes, (2) clipping 10 sequences of 75-100 bp in length from each artificial chromosome, and (3) repeating each sequence 10 times. These steps resulted in a dataset of 1,000 reads and a reference genome consisting of 10 short artificial chromosomes, both of which were used to test the SA_RunStats and SA_Run2Ref workflows. The synthetic dataset was further split in two halves to create Run 1 and Run 2 that were used to test the SA_Run2Run workflow. SeqAssist output results for the synthetic dataset are in agreement with expected results (see files in the SeqAssist software package for details), validating the scripts coded for all three workflows.

Here we demonstrate the applications of SeqAssist to preliminary analysis of multiple experimental NGS datasets. As the SA_RunStats workflow is an integral part of the SA_Run2Run workflow, we focus on the SA_Run2Ref and SA_Run2Run workflows in the following experiments. All the experiments were performed on a Dell M710 Blade server equipped with 283 GB of DDR3 memory at 1,066 MHz speed, an Intel^® ^Xeon^® ^E5630 CPU Quad-core that runs at 2.53 GHz, and two separate hard drives of 1.3 TB and 2.9 TB. The operating system was Red Hat Enterprise Linux Server release 6.3 (Santiago) using the CentOS 64-bit distribution.

### Experimental NGS datasets

We selected multiple NGS datasets and two organisms of contrasting genomic complexity to demonstrate the features of SeqAssist. These datasets represented both fixed and variable length reads generated on Illumina and 454 sequencing platforms, respectively. The chosen organisms were the bacteria *Escherichia coli*, a prokaryote with a simple and small circular genome of 4.6 Mb in length [16], and the water flea *Daphnia pulex*, an eukaryote with a recently published draft genome consisting of 5,191 scaffolds with a total length of ca. 200 Mb [17].

Two datasets obtained from an *E. coli *genome sequencing project were downloaded from http://data.clovr.org/. One dataset (named Ecoli_454_500K) contains 500,000 shotgun 454 titanium sequences (variable length reads in SFF format), and the other (named Ecoli_I4M_R1 and Ecoli_I4M_R2) contains 4,000,000 paired-end shotgun Illumina sequences (2 × 49-bp fixed length reads in FASTQ format). The Bio.SeqIO module in Biopython (http://biopython.org/wiki/Main_Page) was employed to convert the SFF format to the FASTQ format for the 454 dataset by the command $ python -c "from Bio import SeqIO; SeqIO.convert('in.sff', 'sff', 'out.fastq', 'fastq');". The *D. pulex *datasets were collected in-house by repeatedly sequencing two libraries, each of which was prepared from genomic DNA isolated from an individual animal. One animal came from a population named ECT (acronym for "Environmental Consulting & Testing", the vendor from which it was obtained, Superior, WI, USA) and the other from another population named TCO (acronym for "The Chosen One", kindly donated by Dr. Norman D. Yan, York University, Toronto, ON, Canada).

To demonstrate the scalability of SeqAssist, we have also chosen a human genome re-sequencing dataset of the CEU HapMap individual NA12878 at a 15-fold coverage with an insert size of 300 bp and 3.6% duplicate reads. The dataset (SRA ID: ERX012406) consists of 7 paired-end Illumina Genome Analyzer IIx runs and is downloadable from NCBI's SRA database at http://www.ncbi.nlm.nih.gov/sra/?term=ERX012406. The reference diploid human genome (hg18) consists of 23 pairs of chromosomes or 3,234 Mb in total.

### The SA_Run2Ref workflow

**Figure 2:**
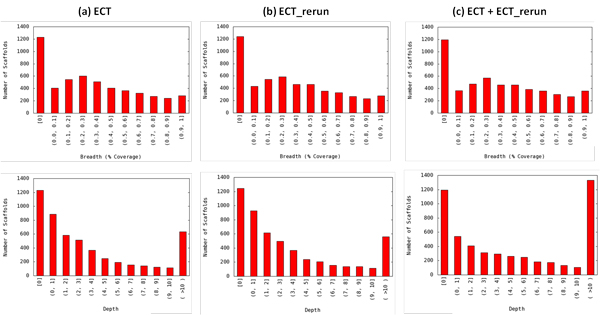
**Distribution of scaffold coverage breadth and depth generated in the output files of the SA_Run2Ref workflow for two genome re-sequencing datasets produced for the same ECT gDNA library and their combination: (a) ECT, (b) ECT_rerun, and (c) ECT + ECT_rerun**. See Table 1 for more information about the sequencing runs. Breadth and depth bins are open at the lower end and closed at the higher end, and breadth is expressed as percentage. For instance, (0.3, 0.4] stands for 30% < breadth ≤ 40%, and (0, 1] stands for 0 < depth ≤ 1.

The ECT *D. pulex *gDNA library was sequenced twice without multiplexing, generating two paired-end sequencing datasets, ECT and ECT_rerun. These two datasets as well as their combined dataset were run through the SA_Run2Ref workflow, producing statistics presented in Table 1. Approximately 88% of the cleaned reads from the ECT or the ECT_rerun dataset was mapped to the reference genome, covering 76% of the 5,191 scaffolds or 64% of the entire genome at a 9-fold depth. The combined dataset covered less than 1% more scaffolds than individual datasets, and it also had similar genome coverage breadth and evenness as the two separate datasets, even though it doubled the genome coverage depth. The distribution of scaffold coverage breadth showed a very similar pattern with ca. 1200 scaffolds uncovered for all three datasets (Figure 2). In comparison with the two separate datasets, the combined dataset covered 830 and 895 more scaffolds at > 4-fold depth or 700 and 774 more at > 10-fold depth than the ECT and the ECT_rerun datasets, respectively (Figure 2). The number of scaffolds with a coverage breadth of 50% or less in the two separate datasets was 188 (ECT) or 218 (ECT_rerun) more than that in the combined dataset. These results indicate that the additional sequencing run (ECT_rerun) did not improve much coverage breadth or evenness, and that the two runs covered almost the same scaffolds.

**Table 1:** Basic statistics produced by SA_Run2Ref for two sequencing run datasets.

Illumina MiSeq runs (read length = 2 × 151 bp)	ECT	ECT_rerun	ECT + ECT_rerun
Total number of raw paired-end reads	7,575,822	7,064,035	14,639,857

Total number of cleaned reads	7,524,261	7,041,454	14,565,715

Total number of reads mapped to reference genome	6,573,572	6,193,164	12,766,736

Mapped/Cleaned reads (%)	87.37	87.95	87.65

Total number of scaffolds in reference genome	5,191	5,191	5,191

Number of covered reference scaffolds	3,960	3,948	3,998

Covered/Total scaffolds (%)	76.29	76.05	77.02

Genome coverage breadth (%)	64.48	64.32	66.12

Genome coverage depth	9.24	8.67	17.91

standard deviation of scaffold coverage depth	96.11	91.88	186.95

average scaffold coverage depth	16.27	15.41	31.33

Genome coverage evenness	6.79	6.86	6.82

Run time (min)	44.6	42.0	81.9

The two datasets of paired-end reads were generated using Illumina MiSeq by sequencing the same genomic DNA (gDNA) library prepared for a water flea (*Daphnia pulex*) from an ECT population. Library preparation involved shearing of extracted gDNA using a Covaris M220 focused-ultrasonicator (Woburn, MA). The average of library insert size distribution was 301 bp.

**Figure 3:**
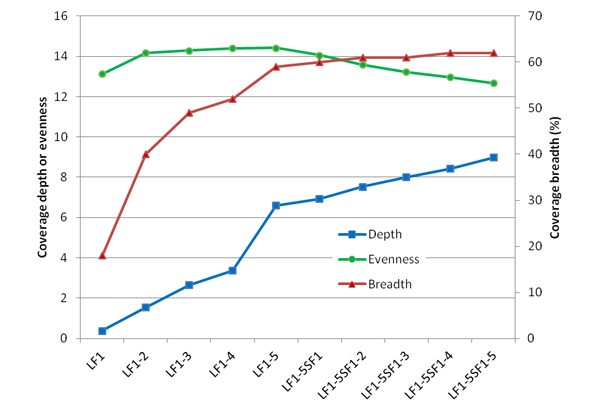
**Change in genome coverage breadth, depth and evenness as more sequencing runs for the same TCO library were pooled and used as the input of SA_Run2Ref**. See Table 2 for the sequencing runs pooled to form reads collections.

**Figure 4:**
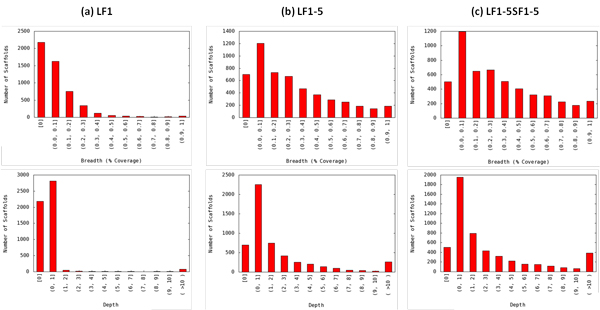
**Change in the distribution of scaffold coverage breadth and depth as more sequencing runs for the same TCO library were pooled and used as the input of SA_Run2Ref**. Shown are distributions for three reads collections: (a) LF1, (b) LF1-5, and (c) LF1-5SF1-5. See Table 2 for the sequencing runs pooled to form reads collections. Breadth and depth bins are open at the lower end and closed at the higher end, and breadth is expressed as percentage. For instance, (0.3, 0.4] stands for 30% < breadth ≤ 40%, and (0, 1] stands for 0 < depth ≤ 1.

The TCO *D. pulex *library was split into two fractions: a large fraction (LF, insert size = 572 bp) and a small fraction (SF, 269 bp). Each fraction was sequenced five times along with 35 other indexed libraries in a multiplexing fashion using Illumina MiSeq, except for the fifth run of LF (LF5) which was pooled with 5 other indexed libraries (Table 2). Hence, the quantity of reads in each LF or SF dataset is equivalent to 1/36 (or 1/6 for LF5) of a MiSeq run. As more datasets were pooled to form new reads collections as input to SA_Run2Ref, the ratio of mapped to cleaned reads remained stable at 82% to 85% (Table 2), and the scaffold coverage evenness had little change (Figure 3). Although the genome coverage depth steadily increased as more runs were added to the reads collection, the genome coverage breadth increased simultaneously until LF5 was added and then reached a plateau (Figure 3). The addition of 2.2 million SF reads raised coverage breadth by only 3% (Table 2 and Figure 3). The change in the distributions of scaffold coverage depth and breadth also supports this conclusion. Except the bin for non-covered scaffolds, the number of scaffolds in every bin increased continuously for both coverage breadth and depth from collection LF1 (Figure 4a) to LF1-5 (Figure 4b), but little difference was observed in the scaffold numbers for coverage breadth between LF1-5 and LF1-5SF1-5 collections (Figure 4c).

**Table 2:** Sequencing datasets and genome mapping of the *Daphnia pulex *TCO library.

Reads collection	Sequencing runs/collection	Library fraction	Raw reads	Cleaned reads	Mapped reads	Mapped/Cleaned reads (%)	Run time (min)	Added run (multiplex, read length)
LF1	LF1	Large only	383,575	381,612	311,919	81.74	7.1	LF1 (36 ×, 2 × 151)

LF1-2	LF1+LF2	Large only	1,083,738	1,076,671	907,601	84.30	13.8	LF2 (36 ×, 2 × 251)

LF1-3	LF1+LF2+LF3	Large only	1,782,006	1,743,523	1,478,140	84.78	21.7	LF3 (36 ×, 2 × 251)

LF1-4	LF1+LF2+LF3+LF4	Large only	2,218,000	2,177,265	1,848,979	84.92	26.1	LF4 (36 ×, 2 × 251)

LF1-5	LF1+LF2+LF3+LF4+LF5	Large only	4,242,048	4,178,856	3,524,528	84.34	45.9	LF5 (6 ×, 2 × 251)

LF1-5SF1	LF1+LF2+LF3+LF4+LF5+SF1	Large + Small	4,542,917	4,478,675	3,766,787	84.10	48.1	SF1 (36 ×, 2 × 151)

LF1-5 SF1-2	LF1+LF2+LF3+LF4+LF5+SF1+SF2	Large + Small	5,084,493	5,014,933	4,204,692	83.84	50.6	SF2 (36 ×, 2 × 151)

LF1-5 SF1-3	LF1+LF2+LF3+LF4+LF5+SF1+SF2+SF3	Large + Small	5,530,560	5,457,878	4,561,648	83.58	52.7	SF3 (36 ×, 2 × 151)

LF1-5 SF1-4	LF1+LF2+LF3+LF4+LF5+SF1+SF2+SF3+SF4	Large + Small	5,920,185	5,845,827	4,872,885	83.36	54.8	SF4 (36 ×, 2 × 151)

LF1-5 SF1-5	LF1+LF2+LF3+LF4+LF5+SF1+SF2+SF3+SF4+SF5	Large + Small	6,411,123	6,333,054	5,270,616	83.22	56.5	SF5 (36 ×, 2 × 151)

All the NGS run datasets were generated by sequencing the TCO gDNA library which was split into two fractions: a large fraction (LF) with an average insert size of 572 bp and a small fraction (SF) with an average insert size of 269 bp. An Illumina MiSeq was used for sequencing and both fractions were each sequenced five times in a 36 × or 6 × multiplexing fashion, resulting in datasets LF1 to LF5 and SF1 to SF5. The reads collections were mapped to a *D. pulex *reference genome by running the SA_Run2Ref workflow.

### The SA_Run2Run workflow

**Figure 5:**
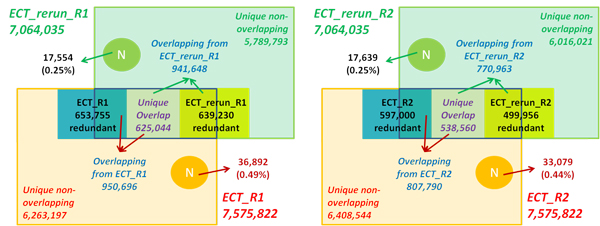
**Breakdown of cleaned reads from two sequencing runs (ECT and ECT_rerun) into overlapping and non-overlapping reads based on the output from SA_Run2Run (see Table 3 for more info)**. "N" represents reads containing N that were removed during preprocessing.

The R1 and R2 files of paired-end sequencing datasets were run separately through the SA_Run2Run workflow. The *E. coli *datasets (Ecoli_454_500K, Ecoli_I4M_R1 and Ecoli_I4M_R2) were split into two halves to create two runs in order to run this workflow (Table 3). For re-sequencing of the *E. coli *genome, output statistics suggest that the paired-end Illumina dataset has a higher redundancy rate than the 454 dataset, which is supported by not only the redundancy within the run (32% to 42% vs. 4%), but also the redundancy of overlapping reads between two split "runs" (97% to 107% vs. 16%) (Table 3). For *D. pulex *genome re-sequencing, the two paired-end datasets had a relatively low redundancy level within each run (8% to 10%) and a low ratio of overlapping reads to total cleaned reads (11% to 13%) (Table 3 and Figure 5). However, based on the output statistics from SA_Run2Ref, the rerun of the ECT library did not improve genome coverage breadth (Table 1 and Figure 2). It can be concluded from output information taken from both workflows that, despite nearly 90% of the reads from the two sequencing runs being different, they essentially cover the same scaffolds. If the main goal of the re-sequencing experiment was to obtain reads representing the entire genome, the additional run of the ECT library was apparently unnecessary.

**Table 3:** The output statistics and derived statistics from running five pairs of NGS datasets through the SA_Run2Run workflow.

NGS datasets	Ecoli_I4M_R1		Ecoli_I4M_R2		Ecoli_454_500K		ECT_R1	ECT_rerun_R1	ECT_R2	ECT_rerun_R2
	**Run1**	**Run2**	**Run1**	**Run2**	**Run1**	**Run2**	**Run1**	**Run2**	**Run1**	**Run2**

**Output statistics**										
Total number of raw reads in the run	2,000,000	2,000,000	2,000,000	2,000,000	250,000	250,000	7,575,822	7,064,035	7,575,822	7,064,035
Total number of cleaned reads in the run	1,968,732	1,997,550	1,999,692	1,999,283	231,123	231,245	7,538,930	7,046,481	7,542,743	7,046,396
Number of unique reads in the run (after removing identical redundant reads)	1,487,552	1,482,834	1,450,704	1,405,779	224,217	224,537	7,114,791	6,702,601	7,242,351	6,839,442
Number of unique reads in the run (after removing identical & inclusive redundant reads)	1,487,552	1,482,834	1,450,704	1,405,779	221,379	221,622	6,885,175	6,407,251	6,945,743	6,546,440
Total number of overlapping reads in the run	712,022	730,780	810,225	834,999	18,304	18,321	950,696	941,648	807,790	770,963
Number of unique overlapping reads in the run	360,898	360,898	403,927	403,927	15,786	15,786	621,978	617,458	537,199	530,419
Number of unique overlapping reads from both runs	360,898		403,927		15,786		625,044		538,561	
**Derived satistics**										
File size after preprocessing	266MB	266MB	266MB	266MB	216MB	216MB	2.6GB	2.4GB	2.5GB	2.4GB
Number of redundant cleaned reads in the run	481,180	514,716	548,988	593,504	9,744	9,623	653,755	639,230	597,000	499,956
Redundancy rate within the run	32.3%	34.7%	37.8%	42.2%	4.4%	4.3%	9.5%	10.0%	8.6%	7.6%
Total number of non-overlapping reads in the run	1,256,710	1,266,770	1,189,467	1,164,284	212,819	212,924	6,588,234	6,104,833	6,734,953	6,275,433
Number of unique non-overlapping reads in the run	1,126,654	1,121,936	1,046,777	1,001,852	205,593	205,836	6,263,197	5,789,793	6,408,544	6,016,021
Number of redundant non-overlapping reads in the run	130,056	144,834	142,690	162,432	7,226	7,088	325,037	315,040	326,409	259,412
Redundancy of non-overlapping reads in the run	11.5%	12.9%	13.6%	16.2%	3.5%	3.4%	5.2%	5.4%	5.1%	4.3%
Number of redundant overlapping reads in the run	351,124	369,882	406,298	431,072	2,518	2,535	328,718	324,190	270,591	240,544
Redundancy of overlapping reads in the run	97.3%	102.5%	100.6%	106.7%	16.0%	16.1%	52.9%	52.5%	50.4%	45.3%
Total overlapping reads/total cleaned reads (each run)	36.2%	36.6%	40.5%	41.8%	7.9%	7.9%	12.6%	13.4%	10.7%	10.9%
Total overlapping reads/total cleaned reads (both runs)	36.4%		41.1%		7.9%		13.0%		10.8%	
**Total runtime (min)**	13.3		13.0		8.2		106.3		110.8	

Overlapping reads are defined as those found in both runs. Unique overlapping reads from both runs are those left after removing redundant reads (identical or inclusive) from the combined overlapping reads from both runs.

With regard to redundant reads, a distinction is drawn between identical and inclusive reads. If reads are of the same length, there is no difference between these two types of redundant reads. Consequently, the number of unique overlapping reads in Run1 is the same as those in Run2 and both runs (see output statistics for Ecoli_I4M_R1 and Ecoli_I4M_R2 in Table 3 for examples). Preprocessing of Illumina datasets may cause length differentiation between cleaned reads. For datasets of reads with variable length, the number of unique reads after removing identical reads is higher than that after removing both types of redundant reads (see output statistics for Ecoli_454_500K, ECT and ECT_rerun datasets in Table 3 and Figure 5). As a result, the number of unique overlapping reads in Run1 may differ from that in Run2 and in both runs (see output statistics for ECT and ECT_rerun datasets in Table 3 and Figure 5).

### Analysis of the human genome re-sequencing dataset

**Figure 6:**
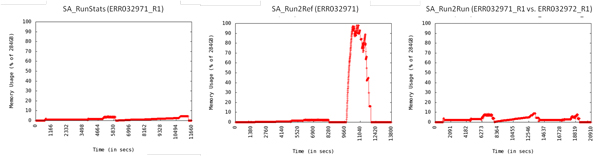
**Memory usage recorded every 5 seconds when running the human genome re-sequencing data through the three SeqAssist workflows**.

Two paired-end sequencing runs, ERR032971 and ERR032972, within the ERX012406 dataset (NA12878) were analyzed using the three workflows to show the scalability of SeqAssist. Both runtime and memory usage were recorded and shown in Table 4 and Figure 6, respectively. The human genome is 15 times bigger than the *D. pulex *genome, and the size of the human genome re-sequencing runs in base pairs (6.4~6.6 Gbp) is nearly triple that of *D. pulex *(2.1~2.2 Gbp, Table 1). However, the runtime of the human data through the SA_Run2Ref workflow (229~319 min, Table 4) was ca. 5.2~7.6 times that of the ECT (44.6 min) or the ECT_rerun (42 min) data (Table 1). For the SA_Run2Run workflow, their runtimes differed only by 3.5-fold, i.e., 96~110 min for *D. pulex *vs. 348~381 min for human. The memory space consumed by SeqAssist when running the two human datasets did not exceed 10% of the 284-GB RAM, except for a surge that occurred when multiple threads were used for calculating the statistics after the completion of BWA alignment at ca. 9700 seconds in the SA_Run2Ref workflow (Figure 6). These results suggest that SeqAssist is fully capable of handling any sequencing run data generated by current NGS platforms for organisms with a reference genome of any size and complexity, and results can be produced rapidly within a working day (less than 8 hours). This feature satisfies the demand for a quick turnaround from mega data to preliminary results.

**Table 4:** Runtime of the three SeqAssist workflows recorded when analyzing two paired-end human genome re-sequencing run data files of a CEU HapMap individual NA12878.

Run data file	Bases (Gbp)	SA_RunStats (min)	SA_Run2Ref (min)	SA_Run2Run (min)
ERR032971_R1	3.2	194	NA	348
ERR032972_R1	3.3	208	NA	
ERR032971_R2	3.2	198	NA	381
ERR032972_R2	3.3	208	NA	
ERR032971	6.4	NA	229	NA
ERR032972	6.6	NA	319	

The read length was 101 bases and the number of reads was 31.9 M and 32.6 M for ERR032971 and ERR032972, respectively. The runtime is rounded up to the closest minute. NA: not applicable.

## Conclusions

We have demonstrated the main features of SeqAssist using multiple genome re-sequencing datasets. Output statistics from SeqAssist can guide the user in evaluating the quality of a DNA library prepared for genome re-sequencing and in deciding whether there is a need to perform additional sequencing runs on the library. Based on the low coverage breadth (66%) and the high reads mapping rate (88%) (Table 1), it appears that the ECT gDNA library may not be a good representation of the entire genome. The same holds true for the TCO library if considering its maximal breadth (62%, Figure 3) and mapping rate (84%, Table 2). In terms of the number of multiplexed sequencing runs required for the TCO library, five runs of the large fraction seemed to be sufficient because they reached the maximal coverage breadth, with a total raw reads number of 4.2 million.

In summary, the SA_RunStats workflow generates basic statistics about an NGS dataset, including numbers of raw, cleaned, redundant and unique reads, redundancy rate, and a list of unique sequences with length and read count. The SA_Run2Ref workflow estimates the breadth, depth and evenness of genome-wide coverage of the NGS dataset at a nucleotide resolution. The SA_Run2Run workflow compares two NGS datasets to determine the redundancy (overlapping rate) between the two NGS runs. Statistics produced by SeqAssist or derived from SeqAssist output files are designed to inform the user: whether, what percentage, how many times and how evenly a genomic locus (i.e., gene, scaffold, chromosome or genome) is covered by sequencing reads, how redundant the sequencing reads are in a single run or between two runs.

SeqAssist is a useful and informative tool that can serve as a valuable "assistant" to a broad range of investigators who conduct genome re-sequencing, RNA-Seq, or *de novo *genome sequencing and assembly experiments. For RNA-Seq experiments, SeqAssist output files that contain unique sequences along with their mapped genomic loci and copy numbers may be readily transformed into gene expression data. An investigator who *de novo *assembles a genome from sequencing data may use SeqAssist to map the original reads to the assembled genome and obtain a ratio of mapped to cleaned reads. As an additional parameter to existing metrics [18], this ratio can be used to objectively compare the quality of different assemblies made from the same sequencing data. For further in-depth analyses of NGS data, one is advised to use other appropriate tools available from the bioinformatics community. For instance, one may choose to apply spliced aligners such as RUM [19] and SpliceSeq [20] to identify splice junctions for alternative splicing detection of RNA-Seq data, or employ a structural variant (SV) discovery software such as BreakDancer [21], Pindel [22] and PRISM [23] to call SV events and discern breakpoints from genome re-sequencing data.

We plan to improve visualization features of SeqAssist in the future versions. Specifically, the nucleotide-resolution mapping and coverage depth (copy number) information generated from SA_Run2Ref shall be transformed into interactive visual graphics to allow the user to visualize gene coverage or expression levels.

## Availability and requirements

• **Project name: **SeqAssist

• **Project home page: **http://orca.st.usm.edu/cbbl/seqassist/

• **Operating systems: **Linux (Ubuntu)

• **Programming language: **Perl v.5.14.2 with the following packages: Parallel::ForkManager and Getopt::Long

• **Other requirements: **BWA (http://bio-bwa.sourceforge.net/), Cutadapt (https://code.google.com/p/cutadapt/), GNUplot, and Java Runtime Environment (JRE version 1.6.0_30 or greater)

• **License: **Free for commercial and academic uses.

• **Any restrictions to use by non-academics: **None.

## Competing interests

The authors declare that they have no competing interests.

## Authors' contributions

PG and CZ conceived the project. PG designed the software architecture. YP implemented the code. NW and PG supervised the coding. ASM developed the GUI. ASM and YP conducted the computational experiments. CZ, NW and PG supervised the experiments. NDB, JGL, AJK and PG generated the *Daphnia pulex *sequencing data. PG, ASM and YP drafted the manuscript. All authors revised, read and approved the manuscript.

## Declarations

Publication charges for this article were funded by the U.S. Army Environmental Quality and Installation Technology Basic Research Program.

This article has been published as part of *BMC Bioinformatics *Volume 15 Supplement 11, 2014: Proceedings of the 11th Annual MCBIOS Conference. The full contents of the supplement are available online at http://www.biomedcentral.com/bmcbioinformatics/supplements/15/S11.
